# M6A Promotes Colorectal Cancer Progression via Regulating the miR-27a-3p/BTG2 Pathway

**DOI:** 10.1155/2023/7097909

**Published:** 2023-02-10

**Authors:** Wenjun Liu, Zilang Zhang, Xitu Luo, Kai Qian, Baojun Huang, Jianzhong Deng, Chengyu Yang

**Affiliations:** ^1^The First Department of General Surgery, The Third Affiliated Hospital of Guangzhou Medical University, Guangzhou 510000, China; ^2^Department of Anorectal Surgery, The First People's Hospital of Foshan, Foshan, Guangdong 528000, China; ^3^The First Department of General Surgery, The First People's Hospital of FoShan, Foshan, Guangdong 528000, China; ^4^Department of Vascular Surgery, Nanfang Hospital, Southern Medical University, Guangzhou 510000, China

## Abstract

Long noncoding (lnc) RNAs regulate cancer progression. However, the importance of lncRNAs and how they are regulated in colorectal cancer (CRC) are unclear. We aim to evaluate the function of lncRNA ADAMTS9-AS2 in CRC and its fundamental mechanism. Levels of ADAMTS9-AS2, miR-27a-3p, and B-cell translocation gene 2 (BTG2) were measured by qPCR. Cell viability was analyzed by CCK-8 and colony formation. Migration and invasion were tested by transwell assay. The interactions among ADAMTS9-AS2, miR-27a-3p, BTG2, and YTHDF2 were analyzed by luciferase test, immunoblotting, RNA pull-down, or RNA immunoprecipitation (RIP). An animal model was adopted to assess ADAMTS9-AS2's function. Overexpressing ADAMTS9-AS2 inhibited cell migration, invasion, colony formation capacity, and proliferation *in vitro*. The direct targeting of miR-27a-3p by ADAMTS9-AS2 abrogated the latter's effect in CRC cells. BTG2 was identified a target of miR-27a-3p, and silencing BTG2 weakened miR-27a-3p's effect. Knocking down ADAMTS9-AS2 abolished sh-YTHDF2's inhibitory effect on cell proliferation and invasion. Finally, overexpressing ADAMTS9-AS2 restrained xenograft growth. M6A reader YTHDF2-mediated degradation of ADAMTS9-AS2 promotes colon carcinogenesis via miR-27a-3p/BTG2 axis.

## 1. Introduction

Colorectal cancer (CRC) is a leading gastrointestinal system's malignancy, and a major reason of tumor-related deaths globally due to increased morbidity. Given the unclear symptoms of early CRC, almost 60% are diagnosed at the advanced stage [1]. A key reason for CRC death is tumor recurrence and metastasis, which is closely linked to migration [2]. Hence, it is critical to better understand CRC's progression and metastasis.

Long noncoding RNAs (lncRNAs) regulate gene expression through various mechanisms [3]. Several lncRNAs have been identified in recent years that regulate tumor progression. A study reported that lncRNA HOTAIRM1 promoted thyroid cancer cells' growth and invasiveness [4]. Furthermore, data showed that the lncRNA FGD5-AS1 promoted chemoresistance of CRC cells [5]. ADAMTS9 is a tumor suppressor, and its antisense RNA 2 (ADAMTS9-AS2) transcript is a lncRNA that may impede tumor progression and metastasis [6]. Wang et al. reported that ADAMTS9-AS2 suppressed gastric tumor cell growth via regulating the expression of SPOP [7]. However, ADAMTS9-AS2's function in CRC is elusive.

N6-methyladenosine (m6A) is a common mRNAs modification [8]. m6A readers, such as YTHDF2, recognize m6A-containing mRNAs to regulate their stability [9]. There is evidence that aberrant m6A modification affects tumorigenesis. For instance, reduced m6A methylation in EC cells suppressed PHLPP2, and increased the positive regulator mTORC2 [10].

MicroRNAs (miRNAs) modulate gene expression through interaction with mRNA's 3′UTR [11]. Data demonstrated that miR-27a-3p promotes CRC cell proliferation and motility [12]. It is also a biomarker for various malignancies and inhibits the tumor suppressor BTG2 [13]. LncRNAs can bind miRNAs as sponges and relieve the inhibitory effect of the latter on target mRNAs. Kong et al. reported that the lncRNA MCF2L-AS1 enhanced CRC cells' EMT via regulating miR-105-5p/RAB22A [14].

Among malignant tumors, the incidence and mortality of lung cancer have always been among the top in the world. Lung cancer is histopathologically divided into non-small-cell lung cancer (NSCLC) and small cell lung cancer. About 85% of lung cancer patients have non-small-cell lung cancer. Advances in the diagnosis and treatment have helped to improve the survival of cancer patients; However, the 5-year survival rate for NSCLC was 17.7. In addition, about 85% of patients with NSCLC are diagnosed at advanced stages. Therefore, further study of the pathogenesis of NSCLC, identification of new therapeutic targets, and prognostic biomarkers are the key to improve patient survival.

In this study, through bioinformatics analysis, the authors first found that RMRP may play an important role in NSCLC. Further studies showed that m6A modification improved the stability of methylated RMRP transcripts by reducing the rate of RNA degradation. In addition, RMRP can promote cell proliferation, migration, and invasion. Mechanistically, RMRP promotes TGFBR1 transcription by recruiting YBX1 to the TGFBR1 promoter. Here, we investigated the function of ADAMTS9-AS2 in CRC progression, and its regulatory influence of m6A modification. Data demonstrated that the novel ADAMTS9-AS2/miR-27a-3p/BTG2 ceRNA regulatory network might regulate CRC progression.

## 2. Materials and Methods

### 2.1. Patient Samples

Seventy-eight paired tumor and adjacent colorectal tissues from CRC patients who underwent surgical resection between February, 2016, and February, 2019, at the 1st People's Hospital of Foshan were included. Patients underwent no radio- or chemotherapy. Informed consents were received. This research has the approval from Ethics Committee of Nanfang Hospital.

### 2.2. Cell Culture and Transfection

Human CRC cells (LoVo, RKO, SW480, HCT116, and HT-29) and normal colon mucosa cells (NCM460) purchased from ATCC were kept in RPMI-1640 with 10% FBS (Gibco) at 37°C. The pcDNA3.1-ADAMTS9-AS2, pcDNA3.1-FTO and pcDNA3.1-YTHDF2 plasmids, the small interfering RNAs (siRNAs) specific for BTG2 (si-BTG2) and ADAMTS9-AS2 (si-ADAMTS9-AS2), and short hairpin (sh) RNA for YTHDF2 (sh-YTHDF2) were obtained from GenePharma (Suzhou). Hsa-miR-27a-3p mimics/inhibitors/negative control (NC) were obtained from GenePharma. Lipo2000 was adopted for transfections.

### 2.3. RT-qPCR

RNAs were isolated using TRIzol (Invitrogen), and cDNA was synthesized with a kit (Takara). qPCR was done with SYBR-Green Mix (ABI). The expression change was calculated by 2^−ΔΔCq^ [15]. Primers are shown as follows:

### 2.4. Methylated RNA Immunoprecipitation (MeRIP) Assay

Methylated RNA immunoprecipitation (MeRIP) is based on the principle of specific antibody specific binding to methylated modified bases and on the basis of RNA immunoprecipitation enrichment of methylated modified fragments, and then through high-throughput sequencing, the results were obtained by studying the RNA regions where methylation occurred on a transcriptome scale. m6A abundance was tested by EpiQuik. m6A RNA methylation quantitative kit (Biovision) was used for the m6A abundance test for the EpiQuik assay. In brief, 250 ng RNA was probed with m6A antibodies. The immunoprecipitation was reverse-transcribed to cDNA, then m6A-lncRNA levels were measured by qRT-PCR.

### 2.5. Immunoblotting

Proteins were isolated by RIPA buffer (Thermo), resolved by 8% SDS-PAGE, and blotted to PVDF membranes (Thermo). After blocking, membranes were probed with anti-YTHDF2 (1 : 3,000; ab220163), anti-BTG2 (1 : 2,000; ab244260), and anti-GAPDH (1 : 5000; ab8245) antibodies at 4°C. After washing, blots were probed with the 2nd antibody.

### 2.6. CCK-8 Analysis

Cells were cultured for 24, 48, 72, and 96 h; then, 10 *µ*l CCK-8 was provided and kept for 2 h. OD470 was recorded with a plate reader.

### 2.7. Transwell Assay

Transwell inserts (8 m, Costar, Corning) with (invasion assay) or without (migration assay) Matrigel (Matrigel Basement Membrane Matrix, Corning) coating were placed in 24-well plates. Cells were cultured in top chambers without serum at a concentration of 0.1 million cells/well (invasion) or 0.5 × 105 cells/well (migration), and bottom chambers were loaded with RPMI-1640 (10% FBS). Two days later, cells on the top surface were discarded, while cells traveled through membranes were fixed and counted.

### 2.8. Colony Formation Assay

Cells were cultured (2 × 10^3^ cells/well) for 8 days. Colonies were fixed by 3.8% PFA, stained with hematoxylin, air-dried, and counted.

### 2.9. Luciferase Reporter Assay

The potential miRNAs that bind to ADAMTS9-AS2 were predicted using DIANA-LncBase Predicted v2, and its downstream target of the candidate miRNAs was predicted by TargetScan. The pmirGLO-ADAMTS9-AS2-wild-type (WT) or pmirGLO-ADAMTS9-AS2-mutant (MUT) reporter plasmids (Sangon Bio) and hsa-miR-27a-3p mimic/inhibitor/NC were cotransfected to CRC cells. Luciferase activity was detected 2 days later (Promega). Cells were cotransfected with WT- or MUT-pmirGLO-BTG2 and miR-27a-3p mimic/inhibitor/NC.

### 2.10. RNA Immunoprecipitation (RIP) Assay

EZ-Magna RIP kit was used. Cells were broken using RIPA buffer and probed with anti-YTHDF2, anti-IgG or anti-Ago2 antibodies, or NC IgG (Abcam, USA). Precipitated RNA was measured by qPCR.

### 2.11. RNA Pull-Down Assay

Lysates of SW480 and HCT116 were incubated with biotin-labeled ADAMTS9-AS2 probe (RiboBio) and streptavidin-coupled magnetic beads. Proteins in the complex pulled down by ADAMTS9-AS2 were analyzed by immunoblotting.

### 2.12. Xenograft Growth Assay

SW480-ADAMTS9-AS2 or control cells were seeded to female BALB/c nude mice. Tumors were monitored every week. Four weeks later, tumors were collected. Expressions of ADAMTS9-AS2, miR-27a-3p, and BTG2 were detected.

### 2.13. Data Analysis

SPSS 22.0 was adopted for analyzing data (mean ± SD). Comparisons between two or more groups were done by *t*-test or ANOVA. *P* < 0.05 was designated as significant.

## 3. Results

### 3.1. ADAMTS9-AS2 Is Decreased in CRC

**Figure 1:**
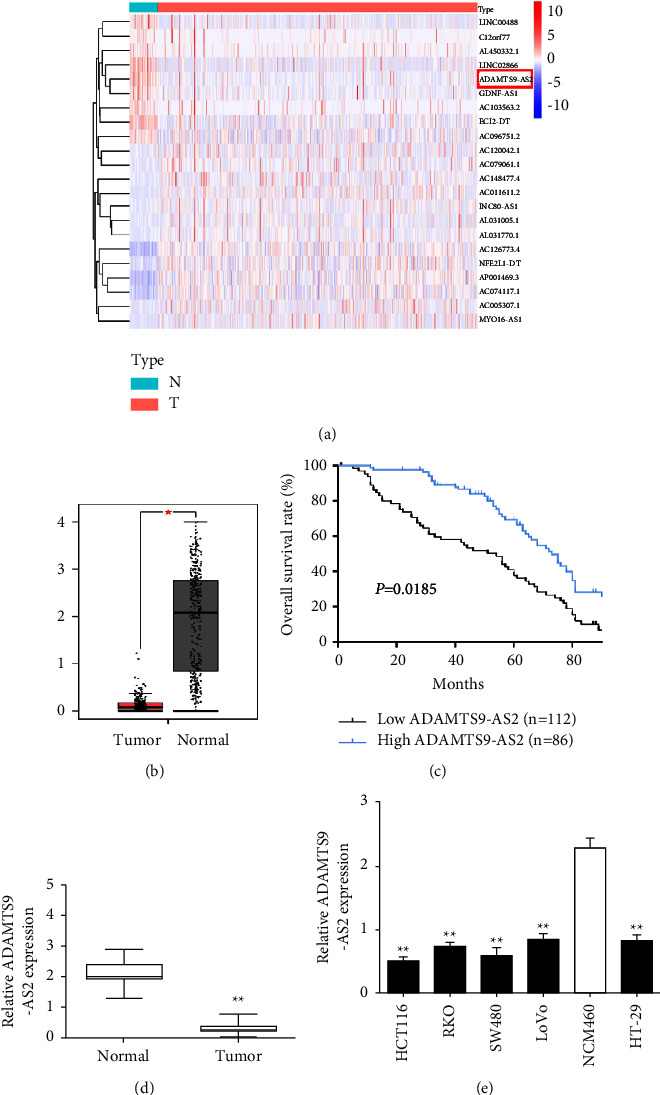
ADAMTS9-AS2 was decreased in CRC. (a) Heatmap of the lncRNA expression. (b) ADAMTS9-AS2 levels from TCGA datasets. (c) Kaplan–Meier analysis of ADAMTS9-AS2 levels and OS. (d) The level of ADAMTS9-AS2. (e) ADAMTS9-AS2 levels in CRC cells. ^*∗*^*P*  <  0.05 and ^*∗∗*^*P*  <  0.01.

Levels of ADAMTS9-AS2 in the CRC and normal colon tissues were analyzed using microarray data downloaded from TCGA database. The heatmap revealed that ADAMTS9-AS2 was drastically downregulated in CRC tissues (Figure 1(a)). In the GEPIA datasets as well, ADAMTS9-AS2 in CRC was considerably decreased than normal colon tissues (Figure 1(b)). Furthermore, patients with lower ADAMTS9-AS2 showed a shorter overall survival (OS) (Figure 1(c)). To verify the in silico data, we analyzed 78 pairs of CRC and adjacent tissues, CRC cells, and colon epithelial cells. ADAMTS9-AS2 was sharply suppressed in the CRC tissue (Figure 1(d)) and cell (Figure 1(e)). Thus, ADAMTS9-AS2 is downregulated in CRC and portends a poor prognosis.

### 3.2. The Effects of ADAMTS9-AS2 Are Partially Returned When Targeted miR-27a-3p Is Highly Expressed on CRC Cells

**Figure 2:**
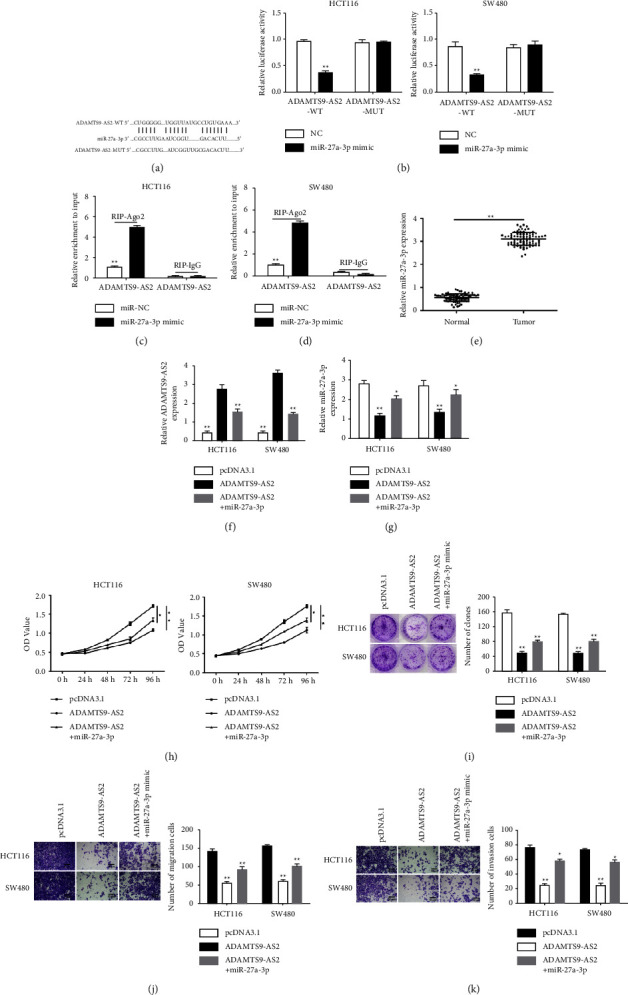
ADAMTS9-AS2-targeted miR-27a-3p returned the effects of ADAMTS9-AS2 on CRC cells. (a) miRNA target of ADAMTS9-AS2. (b–d) ADAMTS9-AS2 interaction with miR-27a-3p. (e) miR-27a-3p in CRC tissues. (f, g) Levels of ADAMTS9-AS2 and miR-27a-3p. (h, i) CCK-8 and clone formation. (j, k) Cell migration and invasion. ^*∗*^*P*  <  0.05 and ^*∗∗*^*P*  <  0.01.

Bioinformatics analysis suggested that ADAMTS9-AS2 binds to miR-27a-3p via complementary base pairing (Figure 2(a)), which was proved by the luciferase assay. The luciferase activity was reduced in HCT116/SW480 cotransfected with miR-27a-3p mimic and pmirGLO-ADAMTS9-AS2-WT, whereas ADAMTS9-AS2-MUT showed no effect. In contrast, the luciferase activity was increased in cells cotransfected with miR-27a-3p inhibitor and pmirGLO-ADAMTS9-AS2-WT but not ADAMTS9-AS2-MUT (Figure 2(b)). The RIP assay further showed significantly higher enrichment of ADAMTS9-AS2 in miR-27a-3p mimic (Figures 2(c) and 2(d)). miR-27a-3p was upregulated in CRC tissues (Figure 2(e)). pcDNA3.1-ADAMTS9-AS2 transfection strikingly downregulated miR-27a-3p, which was partially reversed by miR-27a-3p (Figures 2(f) and 2(g)). CCK-8 and colony formation proved that pcDNA3.1-ADAMTS9-AS2-inhibited cell growth was diminished after miR-27a-3p (Figures 2(h) and 2(i)). The inhibitory effect on cell migration (Figure 2(j)) and invasion (Figure 2(k)) caused by pcDNA3.1-ADAMTS9-AS2 were alleviated by miR-27a-3p.

### 3.3. Knockdown of BTG2 Relieved miR-27a-3p-Silencing-Induced Effects on CRC Cells

**Figure 3:**
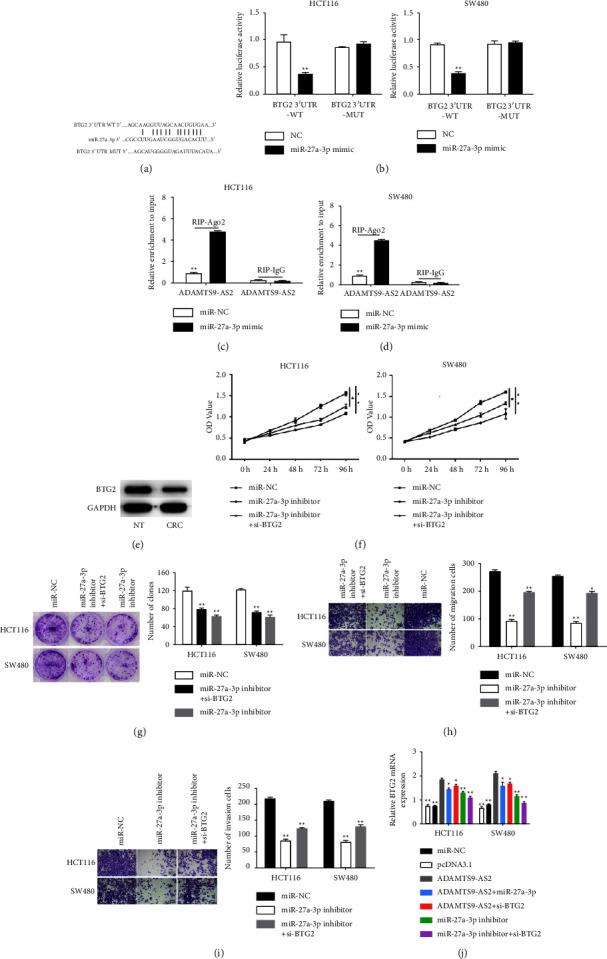
Targeting BTG2 by miR-27a-3p returned miR-27a-3p's inhibition on CRC cells. (a) miR-27a-3p's mRNA target. (b–d) Interaction of ADAMTS9-AS2 and miR-27a-3p. (e) BTG2 levels in CRC tissues. (f, g) CCK-8 and clone formation. (h, i) Cell migration/invasion assay. (j) BTG2 levels after transfection. ^*∗*^*P*  <  0.05 and ^*∗∗*^*P*  <  0.01.

We next identified BTG2 as a miR-27a-3p's target by analyzing 3′-UTR in the Starbase v3.0 database (Figure 3(a)). Transfecting miR-27a-3p and BTG2 3′ UTR-WT suppressed luciferase activity, while no difference was detected in BTG2 3′ UTR-MUT treatment. Transfecting miR-27a-3p inhibitor and BTG2-WT also increased the luciferase activity, whereas no difference was found in BTG2 3′ UTR-MUT treatment (Figure 3(b)). The RIP assay revealed greater enrichment of BTG2 in miR-27a-3p mimic (Figures 3(c) and 3(d)). In addition, BTG2 protein levels were downregulated in CRC tissues (Figure 3(e)). We then overexpressed BTG2 in cells transfected miR-27a-3p, and demonstrated BTG2 reversed miR-27a-3p's effects in cell growth (Figure 3(f)) and colony-forming capacity (Figure 3(g)). Furthermore, miR-27a-3p enhanced the migration (Figure 3(h)) and invasion (Figure 3(h)) and invasion of the CRC cells (Figure 3(i)), which was abrogated by BTG2. Furthermore, overexpressing ADAMTS9-AS2 upregulated BTG2 mRNA in HCT116 and SW480 cells, which was counteracted by miR-27a-3p mimic and siRNA-mediated BTG2 knockdown. Consistent with this, inhibiting miR-27a-3p also upregulated BTG2 mRNA in the CRC cell lines and was neutralized by si-BTG2 (Figure 3(j)). Taken together, ADAMTS9-AS2 may regulate BTG2 levels in CRC cells through an indirect interaction with miR-27a-3p.

### 3.4. YTHDF2 Enhanced the Degradation of m6A-ADAMTS9-AS2 in CRC Cells

**Figure 4:**
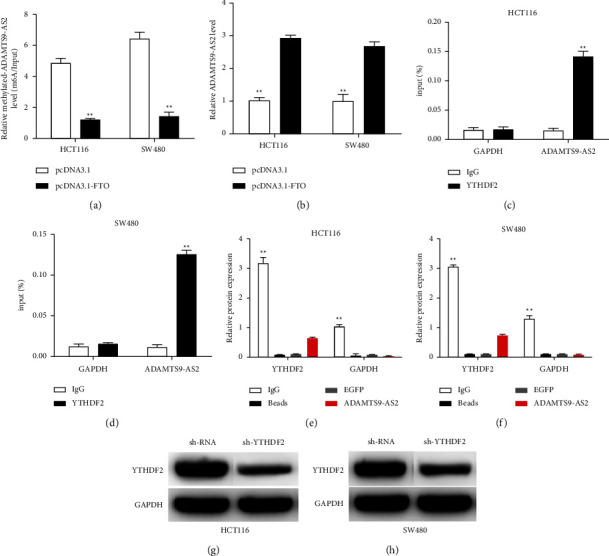
YTHDF2 increased degradation of m6A-methylated ADAMTS9-AS2 in CRC cells. FTO or pcDNA were transfected to HCT116 and SW480. (a) The enrichment of m6A-modified ADAMTS9-AS2 was measured using a MeRIP-qPCR experiment. (b) ADAMTS9-AS2 levels. (c, d) The endogenous combination of ADAMTS9-AS2 and YTHDF2 was measured by RIP and qPCR. (e, f) RNA pull-down confirmed that YTHDF2 recognized ADAMTS9-AS2. (g, h) Western blotting revealed sh-YTHDF2 transfection effectiveness. ^*∗*^*P*  <  0.05 and ^*∗∗*^*P*  <  0.01.

The m6A demethylase FTO was overexpressed in the CRC cell lines. We therefore hypothesized that FTO may affect ADAMTS9-AS2 expression levels in CRC cells by altering its methylation status. As illustrated in Figure 4(a), overexpressing FTO in the SW480 and HCT116 cells significantly reduced m6A-ADAMTS9-AS2 levels. Furthermore, the hypomethylation of ADAMTS9-AS2 was related to a significant increase in its expression levels (Figure 4(b)), indicating that ADAMTS9-AS2 is regulated by m6A modification. In comparison to IgG immunoprecipitation, a RIP experiment demonstrated a higher concentration of ADAMTS9-AS2 in YTHDF2 immunoprecipitation (Figures 4(c) and 4(d)). RNA pull-down indicated that the complex pulled down by ADAMTS9-AS2 contained an abundance of YTHDF2 protein (Figures 4(e) and 4(f)). The results revealed that YTHDF2 recognized ADAMTS9-AS2. YTHDF2 knockdown effectively reduced ADAMTS9-AS2 degradation (Figures 4(g) and 4(h)).

### 3.5. YTHDF2 Silencing Inhibited CRC Cells by Preventing the Degradation of Methylated ADAMTS9-AS2

**Figure 5:**
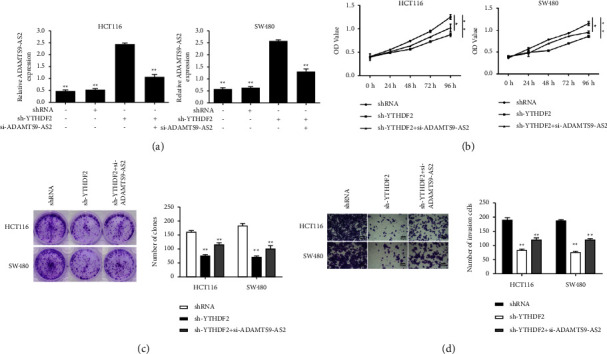
YTHDF2 knockdown suppressed growth and invasion of CRC cells by reducing m6A-modified ADAMTS9-AS2 degradation. (a) Levels of ADAMTS9-AS2. (b, c) Cell proliferation and colony formation assay. (d) Cell invasion assay. ^*∗*^*P*  <  0.05 and ^*∗∗*^*P*  <  0.01.

To assess YTHDF2's function in CRC, we silenced it in cells overexpressing ADAMTS9-AS2. sh-YTHDF2's promotive effect on ADAMTS9-AS2 level was inhibited by si-ADAMTS9-AS2 (Figure 5(a)). YTHDF2 knockdown inhibited cell growth (Figure 5(b)), colony formation (Figure 5(c)), and invasion (Figure 5(d)), and the malignant phenotype was rescued by ADAMTS9-AS2 silencing.

### 3.6. ADAMTS9-AS2 Prevents Tumor Growth

**Figure 6:**
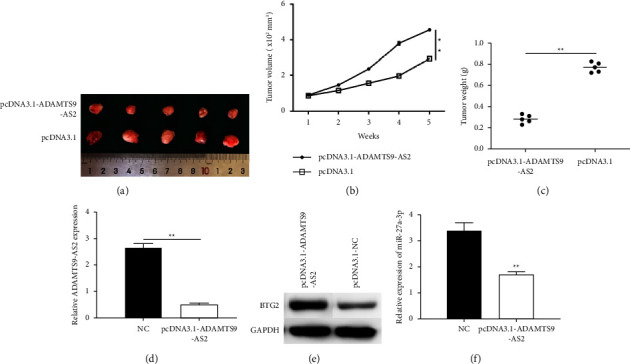
ADAMTS9-AS2 overexpression retarded tumor growth. SW480-pcDNA-ADAMTS9-AS2 or control cells were inoculated to nude mice for four weeks. (a, b) Tumor volume. (c) Tumor weight. (d) ADAMTS9-AS2 levels. (e) BTG2 protein levels. (f) miR-27a-3p levels in xenografts. ^*∗*^*P*  <  0.05 and ^*∗∗*^*P*  <  0.01.

The suppressive effects of ADAMTS9-AS2 on CRC growth was also evaluated by establishing xenografts. The CRC cells overexpressing ADAMTS9-AS2 showed less tumor volume (Figures 6(a) and 6(b)) and weight (Figure 6(c)) compared to controls, indicating that ADAMTS9-AS2 suppressed tumor growth. The ADAMTS9-AS2 levels were reduced in the tumor tissues (Figure 6(d)). Moreover, the overexpression of ADAMTS9-AS2 upregulated BTG2 (Figure 6(e)) and downregulated miR-27a-3p in CRC (Figure 6(f)).

## 4. Discussion

Increasing evidence shows that aberrant lncRNAs are linked to the development of CRC. For example, ENO1-IT modulates KAT7 histone acetyltransferase and consequently altered CRC biological function [16]. Furthermore, LINC00265 is upregulated in CRC, and its knockdown in mice significantly reduced colorectal carcinogenesis [17]. We found that ADAMTS9-AS2 was decreased in the CRC samples in TGCA database, and associated with the worse survival rate. ADAMTS9-AS2 (Ensembl, ENSG00000241684) has been linked to several tumor-associated genes in multiple cancers. For example, ADAMTS9-AS2 was increased in TMZ-resistant glioblastoma cells to enhance chemoresistance by upregulating the FUS/MDM2 axis [18]. We also demonstrated that ADAMTS9-AS2 decreased in CRC. Decreased ADAMTS9-AS2 was linked to poor differentiation, lymph node metastases, and advanced TNM staging. Overexpressing ADAMTS9-AS2 in CRC cells inhibited their malignant potential *in vitro*. Thus, ADAMTS9-AS2 is a potential marker for the CRC prognosis and may function as a tumor suppressor.

The hypothesis that ceRNA (competitive endogenous RNA), proposed by Pier Paolo Pandolfi's group at Harvard Medical School in 2011, is a mode of regulating gene expression. Transcripts that share miRNA-binding sites compete to bind the same miRNA, thereby regulating each other's expression levels. Based on ceRNA hypothesis, lncRNAs regulate mRNAs post-transcriptionally by competitively binding to miRNAs containing response regions [19, 20]. For instance, lncRNA UCA1 sponges miR-143 and upregulates MYO6, thereby promoting CRC [21]. Likewise, UICLM promotes CRC metastasis by upregulating ZEB2 via its sponging action on miRNA-215 [22]. A previous study showed that miR-27a-3p was upregulated in CRC, and silencing it decreased cell growth [12].

BTG2 was proved as a miR-27a-3p's target by the luciferase assay and RIP assay. BTG2 regulates cell division, DNA repair, transcriptional control, and mRNA stability [23]. BTG2 was decreased in different cancers. For instance, BTG2 decrease promoted breast cancer's metastasis [24]. Likewise, miR-6875-3p affects cancer cells' invasiveness via BTG2 [25]. We revealed that BTG2 was decreased in CRC, and its silencing abrogated miR-27a-3p's pro-oncogenic effects. Given the abundance of m6A modification in eukaryotic mRNAs, it has received considerable attention as a regulatory factor in cancer and other pathological and developmental states.

Studies demonstrated that m6A binding protein YTHDF2 destabilized EGFR via binding to m6A site, and inhibited hepatocellular carcinoma cells [26]. YTHDF2 also regulates the stability of lncRNAs and mRNA during cancer development. Another study revealed that YTHDF2 inhibited lncRNA GAS5 in cervical cancer cells by promoting its degradation [27]. Consistent with this, hypermethylation of ADAMTS9-AS2 increased its degradation through the recruitment of YTHDF2. These findings show that aberrant m6A modification of ADAMTS9-AS2 is reliant on YTHDF2. Knocking down YTHDF2 inhibited CRC cell proliferation and invasion by restoring ADAMTS9-AS2 expression, implying that YTHDF2 may have an oncogenic role in CRC.

## 5. Conclusions

Our data indicated miR-27a-3p as a direct target of ADAMTS9-AS2 for the first time. Overexpression of ADAMTS9-AS2 downregulated miR-27a-3p. miR-27a-3p was increased in HT-29/SW480, and its overexpression ameliorated its inhibitory effects of elevated ADAMTS9-AS2. The results indicated that ADAMTS9-AS2 may inhibit CRC through regulating miR-27a-3p. In summary, ADAMTS9-AS2 is downregulated in CRC, and its overexpression inhibited growth and invasion of CRC cells. Mechanistically, ADAMTS9-AS2 functions as a ceRNA against miR-27a-3p, which upregulates BTG2. Furthermore, aberrant m6A modification was associated with the decreased levels of ADAMTS9-AS2 in CRC. The YTHDF2/ADAMTS9-AS2/miR-27a-3p/BTG2 modulatory network is a novel pathway participated in CRC development, and ADAMTS9-AS2 may function as a novel therapeutic target.

## Figures and Tables

**Table 1:** Oligonucleotide sequences.

Name	Sequence (5′-3′)
ADAMTS9-AS2	F: TCTGTTGCCCATTTCCTACC
	R: CCCTTCCATCCTGTCTACTCTA
miR-27a-3p	F: CTAATCGTGTTCACAGTGGCTAAG
	R: TATGGTTTTGACGACTGTGTGAT
BTG2	F: GCGCGGGCTCTTCCTCTTTG
	R: AAGGAAGGCTGGAAGAGTGC
GAPDH	F: TGTTCGTCATGGGTGTGAAC
	R: ATGGCATGGACTGTGGTCAT
U6	F: GCATCTGCAACACTTATCCTATAAT
	R: CGATTCGCGCATATGCTTGTGAT

## Data Availability

The experimental data used to support the findings of this study are available from the corresponding authors upon request.
